# Effect of Major Depressive Disorder on Stroke Risk and Mortality: A Systematic Review

**DOI:** 10.7759/cureus.40475

**Published:** 2023-06-15

**Authors:** Aujala Irfan Khan, Baraa Abuzainah, Sai Dheeraj Gutlapalli, Dipabali Chaudhuri, Kokab Irfan Khan, Roba Al Shouli, Akhil Allakky, Asila A Ferguson, Pousette Hamid

**Affiliations:** 1 Research, California Institute of Behavioral Neurosciences & Psychology, Fairfield, USA; 2 General Practice, California Institute of Behavioral Neurosciences & Psychology, Fairfield, USA; 3 Internal Medicine, California Institute of Behavioral Neurosciences & Psychology, Fairfield, USA; 4 Pediatrics, California Institute of Behavioral Neurosciences & Psychology, Fairfield, USA; 5 Psychiatry, California Institute of Behavioral Neurosciences & Psychology, Fairfield, USA; 6 Neurology, California Institute of Behavioral Neurosciences & Psychology, Fairfield, USA

**Keywords:** icd-10, dsm-5, stroke mortality, stroke risk factor, major depressive disorder, stroke

## Abstract

This study aimed to systematically review the available data on major depressive disorder (MDD) and provide insight into how it may affect stroke risk and mortality. We conducted this systematic review drawing upon research published between July 2002 and July 2022 from the following databases: PubMed, ScienceDirect, and Google Scholar. After eliminating duplicates, screening the title and abstract, determining eligibility, and quality assessment, eight articles were left for utilization in this systematic review (one meta-analysis and seven non-randomized studies). There was a potentially significant association between MDD and stroke risk and mortality. The apparent connection between MDD and stroke has medical and public health relevance, given the high incidence, prevalence, and financial burden of MDD and stroke in the general populace. Therefore, it is imperative that further studies are conducted to confirm and validate this association between MDD and stroke while also elucidating the mechanism involved, investigating potential variables influencing this association, and contrasting MDD with conventional stroke risk factors to determine its predictive usefulness in comparison to traditional risk factors. This will have a significant effect on clinical practice since the information provided by such research will help guide essential targets for stroke prevention.

## Introduction and background

Major depressive disorder (MDD), also known as unipolar major depression, is diagnosed according to the criteria in the American Psychiatric Association's Diagnostic and Statistical Manual of Mental Disorders, Fifth Edition (DSM-5) or the World Health Organization's International Classification of Diseases-10th Revision (ICD-10) [1,2]. A major depressive episode (MDE) is an interval of at least two or more weeks in which a person displays MDD symptoms according to DSM-5/ICD-10 criteria [1,2]. An individual must have one or more MDEs that cannot be explained by another disorder and no history of a hypomanic or manic episode in order to be diagnosed with MDD [1,2]. About 3.76% of the global population has depression, which is around 280 million people worldwide [3]. In 2020, the prevalence of MDEs in the United States was 8.4% in adults (approximately 21 million people) [4]. Moreover, the additional economic cost of MDD in the United States increased by 37.9% over eight years (2010-2018), from $236.6 billion to $326.2 billion [5].

Stroke is one of the most common causes of severe long-term disability and mortality [6,7]. One fatality out of every 19 in the United States was caused by a stroke in 2019 [7]. Moreover, in the United States, every 3.5 minutes, someone dies because of a stroke [7]. Additionally, between 2017 and 2018, stroke-related expenditures in the United States totaled around $53 billion [7]. Over the past decade (2010-2019), the rate of decrease in age-standardized stroke incidence, mortality, and disability-adjusted life-year rates worldwide were substantially slower than over the prior decade (2000-2009) [8]. Better management of current risk factors, as well as the recognition and management of emerging risk factors, is necessary for future decreases in the incidence of stroke [9]. The carotid substudy of the Northern Manhattan investigations revealed that conventional risk factors only account for a small proportion of the variation in carotid plaque, indicating a potential role for unaccounted unconventional risk factors in the formation of atherosclerotic plaque [10,11].

An increasing amount of research points to a bi-directional relationship between depression and stroke [12]. Recent meta-analysis data have implied that common genetic pathways may be a factor in the comorbidity of MDD and stroke, specifically methylenetetrahydrofolate reductase (MTHFR) and apolipoprotein E (ApoE) genes [12]. Moreover, a Mendelian randomization investigation discovered a potential link between MDD and an elevated risk of small vessel stroke [13].

Although numerous studies have found a link between depression and stroke, their interpretations have some flaws [14]. First of all, instead of relying on a clinical diagnosis of MDD, the majority of research evaluates depression based on self-reported questionnaires using depression rating scales [15,16]. Most symptom severity ratings used in depression screening do not give a diagnosis, cannot identify which depressive disorder is present, and can add misclassification bias to the data [17]. Second, most of the research falls short of reporting data on antidepressant use and the treatment results for depression [14]. Even though antidepressant therapies may improve stroke outcomes, some antidepressants, such as selective serotonin reuptake inhibitors (SSRIs), may increase bleeding propensity by blocking platelet aggregation and have been linked to a higher stroke risk [18-21].

Therefore, we conducted this systematic review to explore the impact of MDD on stroke risk and mortality.

## Review

Method

Preferred Reporting Items for Systematic Review and Meta-Analysis (PRISMA) 2020 guidelines were used for this systematic review [22].

Databases and Keywords Used

PubMed, ScienceDirect, and Google Scholar were the databases accessed on July 15, 2022, to conduct research for this systematic review. 

Medical Subject Headings (MeSH) keywords used were: ("Depressive Disorder, Major/analysis" [Majr] OR "Depressive Disorder, Major/anatomy and histology" [Majr] OR "Depressive Disorder, Major/classification" [Majr] OR "Depressive Disorder, Major/complications" [Majr] OR "Depressive Disorder, Major/diagnosis" [Majr] OR "Depressive Disorder, Major/diagnostic imaging" [Majr] OR "Depressive Disorder, Major/epidemiology" [Majr] OR "Depressive Disorder, Major/etiology" [Majr] OR "Depressive Disorder, Major/genetics" [Majr] OR "Depressive Disorder, Major/immunology" [Majr] OR "Depressive Disorder, Major/mortality" [Majr] OR "Depressive Disorder, Major/pathology" [Majr] OR "Depressive Disorder, Major/physiology" [Majr] OR "Depressive Disorder, Major/physiopathology" [Majr] OR "Depressive Disorder, Major/prevention and control" [Majr] OR "Depressive Disorder, Major/psychology" [Majr] OR "Depressive Disorder, Major/statistics and numerical data" [Majr]) AND ("Stroke/analysis"[Majr] OR "Stroke/anatomy and histology" [Majr] OR "Stroke/complications" [Majr] OR "Stroke/diagnosis" [Majr] OR "Stroke/diagnostic imaging" [Majr] OR "Stroke/epidemiology" [Majr] OR "Stroke/etiology" [Majr] OR "Stroke/genetics" [Majr] OR "Stroke/immunology" [Majr] OR "Stroke/mortality" [Majr] OR "Stroke/pathology" [Majr] OR "Stroke/physiology" [Majr] OR "Stroke/physiopathology" [Majr] OR "Stroke/prevention and control" [Majr] OR "Stroke/psychology" [Majr] OR "Stroke/statistics and numerical data"[Majr])

Keywords used for ScienceDirect and Google Scholar: Major depressive disorder and stroke

Inclusion Criteria

Full-text papers published between July 2002 and July 2022 in English, focused on the adult population aged ≥18 years old, and relevant to the question were included.

Exclusion Criteria

Papers discussing the pediatric population, unpublished literature, grey literature, abstract-only papers, or non-English language papers were excluded from this systematic review.

Quality Appraisal

All meta-analysis papers satisfying ≥70% of Assessing the Methodological Quality of Systematic Reviews (AMSTAR) checklist questions were included.

Only moderate (4-6 Newcastle-Ottawa scale (NOS) score) and low-risk (7-9 NOS score) non-randomized studies were included, while high-risk (0-3 NOS score) studies were excluded.

The quality assessment tools stated in Table 1 were used for the appropriate study type.

**Table 1:** Study type and the quality assessment tool used AMSTAR: Assessing the Methodological Quality of Systematic Reviews; NOS: Newcastle-Ottawa scale

Study Type	Quality Assessment Tool	Number of Studies
Meta-analysis	AMSTAR Checklist	1
Non-randomized studies	NOS	7

Results

**Figure 1:**
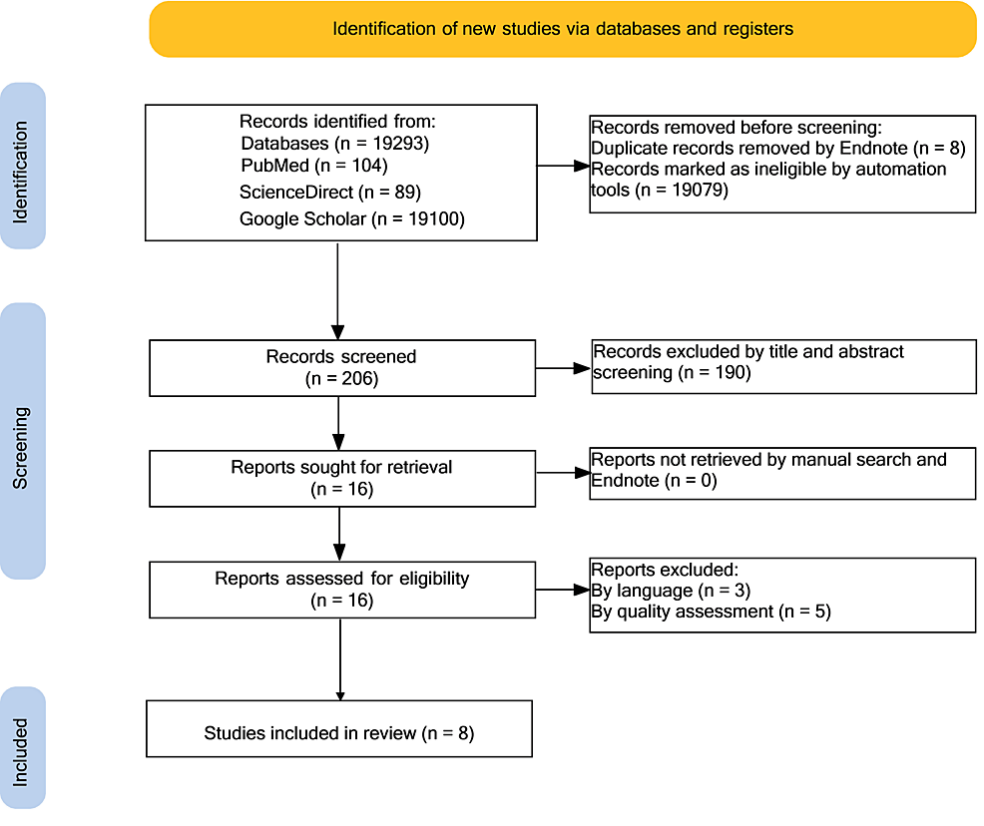
Preferred Reporting Items for Systematic Review and Meta-Analysis (PRISMA) flowchart n = number of records

A total of 19,293 results were identified from the aforementioned databases, of which 19,087 results were removed because of duplicates (eight results) and automation tools (19,079 results). A further 190 results were excluded by screening the title and abstract. The 16 remaining papers were sought for retrieval and evaluated for eligibility. Eight were removed after screening for English-only papers and quality assessment. As a result, eight articles were left for use in this systematic review (one meta-analysis and seven non-randomized studies). The PRISMA flowchart (Figure 1) reveals the filtering process.

Table 2 concisely summarizes all the studies in this systematic review.

**Table 2:** Brief outline of the studies included in this systematic review HIV: human immunodeficiency virus; MDD: major depressive disorder; NA: Not applicable; MDE: major depressive episode.

Author and Year of Publication	Location	Study Type	Purpose of Study	Number of Patients/Studies	Conclusion
Sico et al., 2021 [23]	United States	Prospective	To determine whether depression affects the likelihood of incident ischemic stroke in HIV patients.	106,333	HIV-positive individuals who suffer from depression have a higher risk of ischemic stroke.
Li et al., 2019 [24]	Taiwan	Retrospective	To determine if MDD increases the risk of dying from stroke in patients receiving nonsurgical treatment.	91,120	MDD was not linked to death in stroke patients who had nonsurgical therapy.
Ho et al., 2016 [25]	Singapore	Prospective	To examine the relationships between major and subthreshold depression and mortality from all-cause, cardiovascular disease, and stroke, and to assess to what degree health behavior, medical comorbidity, and functional disability can account for the relationships.	1,070	Both were linked to higher mortality rates, largely due to risky behaviors and physical comorbidities. The only condition independently linked to increased cardiovascular disease and stroke mortality was MDD.
Sun et al., 2016 [26]	China	Prospective	To investigate the impact of MDE on the incidence of stroke and determine whether there may be a dose-response relationship between the number of depression symptoms and ensuing stroke risk in the Chinese population.	487,377	MDE increases the risk of stroke, notably among smokers.
Hamano et al., 2015 [27]	Sweden	Prospective	To assess the relationship between MDD and stroke after adjusting for possible confounding factors and to evaluate whether MDD has a differential effect on stroke in different populations.	326,229	MDD increases the risk of stroke, and gender affects the relationship between MDD and stroke.
Barlinn et al., 2015 [28]	NA	Meta-analysis	To investigate whether patients with depression, who are healthy in terms of their cardiovascular and cerebrovascular systems, are more likely to experience a stroke or whether this link might actually be the result of reverse causality.	28 studies	The link between depression and a higher risk of stroke does not seem to be caused by either cardiac or cerebrovascular diseases.
Thomson et al., 2014 [29]	United Kingdom	Prospective	To investigate the relationship between depression and stroke mortality.	685	Depression patients are more likely to die of a stroke.
Li et al., 2012 [14]	Taiwan	Prospective	To determine whether a history of MDD would raise the risk of stroke directly or indirectly through established stroke risk factors like major metabolic illnesses and to assess whether depressive symptoms or antidepressants are linked to stroke incidence.	5,015	MDD was indirectly linked to a greater risk of future stroke.

Discussion

Link Between MDD and Stroke: Direct or Indirect Interaction?

A 2012 prospective study by Li et al. determined whether a history of MDD would raise the risk of stroke directly or indirectly through established stroke risk factors [14]. A total of 5,015 participants (MDD group = 1,003 and control group = 4,012) were followed up for nine years [14]. Compared to the control group, MDD patients experienced considerably more emergency department visits (p < 0.001), psychiatric hospitalizations (p < 0.001), and suicide attempts (p < 0.001) [14]. MDD patients also experienced higher rates of non-psychiatric hospitalizations (p < 0.001), hypertension (p = 0.016), and hyperlipidemia (p < 0.001) than the control group [14]. In addition, the development of major metabolic illnesses (hypertension, hyperlipidemia, and diabetes mellitus) strongly mediated the increased incidence of stroke in the MDD participants, according to the mediation regression analysis [14].

Ho et al. led a prospective study to examine the relationships between major and subthreshold depression, and mortality from all-cause, cardiovascular disease, and stroke [25]. A total of 1,070 patients ≥60 years old were enrolled between 2003 and 2004 and followed up until mortality (2005-2012) [25]. There were 54 MDD and 101 subthreshold depression participants at baseline [25]. The median follow-up was 9.5 years [25]. MDD and subthreshold depression were linked to higher mortality rates, which were primarily because of risky behaviors (alcohol, sedentary lifestyle, and smoking) and physical comorbidity (heart disease, hypertension, diabetes, stroke, chronic pulmonary disease, multiple comorbidities, and activity of daily living disability) [25]. However, only MDD was associated with stroke mortality and increased cardiovascular disease [25].

In contrast, Barlinn et al. conducted a meta-analysis to investigate whether patients with depression, who are healthy in terms of their cardiovascular and cerebrovascular systems, are more likely to experience a stroke or whether this link might actually be the result of reverse causality [28]. Of the 28 studies (681,139 patients) they evaluated, only seven studies ascertained depression using DSM or ICD criteria [28]. They concluded that the link between depression and a higher risk of stroke does not seem to be caused by either silent or clinically apparent cardiovascular or cerebrovascular disorders [28]. However, a majority of the studies in this meta-analysis employed self-reported questionnaires, introducing misclassification and measurement bias [28].

Effect of MDD on Stroke Risk

Li et al. (2012) reported that patients in the MDD group had significantly greater rates of stroke (4.3% versus 2.8%, p < 0.05) [14]. Similarly, Barlinn and colleagues found that depression was linked to a greater risk of incident stroke (relative risk (RR) = 1.43, 95% confidence interval (CI): 1.19-1.72; p < 0.0001) [28].

The prospective study by Hamano et al. assessed the relationship between depression and stroke risk [27]. They had 326,229 patients; 307,795 patients were in the control group, while 18,434 had MDD [27]. Over the three years of follow-up, 4,718 strokes occurred (control group = 4,434 and MDD group = 284) [27]. Hamano and colleagues found that even after accounting for any potential confounding variables, MDD was linked to a substantially greater risk of stroke (odds ratio (OR) = 1.22, 95% CI: 1.08-1.38) [27].

Sun et al. conducted a prospective study to investigate the impact of MDE on the incidence of stroke [26]. They enrolled 487,377 participants; 2,988 had past year MDE, while 484,389 had no history of MDE (control group) [26]. Median follow-up was 7.2 years [26]. During the follow-up period, there were 27,623 strokes (hemorrhagic = 5,255, ischemic = 21,427, and unknown stroke type = 941); 183 were in the MDE group, and 27,440 were in the control group [26]. In comparison to the control group, those with MDE were more likely to be younger, female, living in rural areas, single, and with a decreased level of education, household income, and BMI; but they were less likely to be physically active, regular smokers, or drinkers [26]. Additionally, those in the MDE group had higher rates of diabetes, while the control group had higher rates of hypertension [26]. Sun et al. found that past year MDE was linked to a 15% increased stroke risk after adjusting for all other variables (adjusted hazard ratio (HR) = 1.15, 95% CI: 0.99-1.33) [26]. They also noted that stroke type (hemorrhagic stroke or ischemic stroke) did not show a statistically significant association with the depression and stroke incidence relationship [26].

Moreover, Sun and colleagues discovered that the number of depressive symptoms and the risk of stroke had a statistically significant dose-response relationship (p = 0.011) [26]. They found that more symptomatic people (especially those who had six (adjusted HR = 1.33) or seven (adjusted HR = 1.47) symptoms) had a greater stroke risk compared to those who had 0-2 (adjusted HR = 1.00) symptoms of depression [26]. Given that the number of symptoms reflects how severely depressed the individual is, it makes sense that, in this study, the risk of stroke could rise along with the severity [26]. Similarly, Li and colleagues (2012) found that stroke incidence was preceded by greater depression severity (assessed by measuring antidepressant refractoriness level and the number of psychiatric visits six months before stroke (psychiatrists are easily available in Taiwan, so increased visits meant higher depression severity)) but not by greater antidepressant use [14].

Sico et al. conducted a prospective study to determine the effect of depression on the risk of incident ischemic stroke in HIV (human immunodeficiency virus) patients [23]. A total of 106,333 patients (HIV-positive = 33,528 and HIV‐negative = 72,805) were enrolled [23]. They were further divided into the following four groups: HIV-positive with depression = 6,554, HIV‐positive without depression = 26,974, HIV‐negative with depression = 13,713, and HIV‐negative without depression = 59,092 [23]. Median follow-up was 9.2 years [23]. HIV-positive with depression group had the highest incident ischemic stroke rates, while the HIV-negative without depression group had the lowest rates [23]. Moreover, after adjusting for sociodemographic traits, cerebrovascular disease risk factors, and HIV-specific factors, individuals with depression and HIV had the highest risk of ischemic stroke (18%) compared to those with only one or none of these conditions [23]. However, adjusting for cocaine and alcohol use disorders, separately and together, decreased the connection between depression and the risk of stroke (no statistical significance noted), indicating that these factors may be partially responsible for the relationship between depression and incident ischemic stroke [23]. Also, patients were classified as having depression if they received a diagnosis of MDD or dysthymic disorder, according to the International Classification of Diseases, Ninth Revision (ICD-9) [23]. However, it is unclear how many had MDD or dysthymia and what effect either disorder had on ischemic stroke risk individually [23].

Effect of MDD on Stroke Mortality

Sico et al. found that of the four groups, HIV-positive with depression group had the worst rates of stroke-free survival (p = 0.001) [23]. Also, Ho et al. discovered that MDD was substantially linked to stroke mortality and increased cardiovascular disease (2.1 times increased risk, 95% CI: 1.07-4.11, p = 0.03) [25]. Their finding is significant for MDD patients ≥60 years old [25].

A 2019 retrospective study by Li et al. determined whether MDD increases the risk of dying from a stroke in people receiving nonsurgical treatment [24]. They enrolled 91,120 patients (MDD group = 18,224 and control group = 72,896) between 1999 and 2005 [24]. During 2006-2012, the number of patients in the MDD group who had a stroke and underwent nonsurgical treatment = 2,265, and patients in the control group who had a stroke and underwent nonsurgical treatment = 712 [24]. All stroke patients who had nonsurgical treatment were followed up for seven years [24]. Li et al. noted that age ≥65 years (HR = 4.35, p < 0.0001), being male (HR = 1.26, p = 0.006), having diabetes mellitus (HR = 1.29, p = 0.0049), Charlson Comorbidity Index scores (CCISs) ≥2 (HR = 1.35, p = 0.0045), and experiencing a hemorrhagic stroke (HR = 2.48, p < 0.0001) were risk factors for mortality in stroke patients with nonsurgical treatments [24]. Moreover, stroke patients in the MDD group who underwent nonsurgical treatment had higher CCISs, had high or low socioeconomic status, were significantly more likely to have coronary heart disease, and were female compared to stroke patients in the control group who underwent nonsurgical treatment [24]. Also, in stroke patients receiving nonsurgical treatment, neither MDD (HR = 1.16, p = 0.1518) nor the level of urbanization (suburban: HR = 0.867, p = 0.1676; rural: HR = 1.199, p = 0.1666) was a risk factor for mortality [24]. The lack of association between MDD and stroke prognosis could be because stroke patients in the MDD group with a high socioeconomic status possibly have access to more medical care, leading to improved stroke prognoses [24]. Another reason could be the exclusion of patients who had surgical treatment for stroke as often following surgery, patients with massive strokes have terrible prognoses [24].

A prospective study by Thomson investigated the relationship between depression and stroke mortality [29]. A total of 685 participants were diagnosed with depression (endogenous depression = 480 and reactive depression = 205) and were followed up for 40 years [29]. The risk of subsequent stroke mortality was higher in males than in women with clinical depression [29]. Moreover, among men with diagnoses of endogenous depression but not among those with diagnoses of reactive depression, death by stroke was a statistically significant cause of death [29]. Furthermore, over time, the correlation between depression and stroke mortality became more significant [29].

Potential Factors Affecting the MDD-Stroke Relationship

Hamano et al. found that the effect of depression on stroke risk was greater in men than in women (OR difference between men and women was 1.30, 95% CI: 1.01-1.68), while Thomson showed that the effect of depression on stroke mortality was more significant in men; indicating that the relationship between depression and stroke was altered by gender [27,29]. 

Sico et al. discovered that baseline antidepressant use, cocaine use, and alcohol use disorders contributed to some of the observed stroke risk, but that combined or individual antiretroviral treatment and HIV-specific variables did not affect the association between depression and ischemic stroke [23]. Future studies should focus on cocaine usage, alcohol use disorders, and antidepressant use to evaluate how treating substance abuse and managing MDD may decrease the likelihood of stroke.

Moreover, Sico and his colleagues found that the depression and ischemic stroke risk relationship was apparent in those <60 years but not in those >60 years, while Sun et al. found the MDD-stroke relationship in <50 years but not in those >50 years; suggesting that the relationship between depression and incident stroke may be more significant in younger people [23,26].

Sun et al. observed that smokers in the MDE group had a 1.71-fold greater stroke risk (adjusted HR = 1.71, 95% CI: 1.31-2.24, p < 0.001) when compared to smokers in the control group [26]. This suggests that the impact of these two risk factors on stroke risk could be influenced by each other, and additional research may provide crucial insights into these interactions.

Limitations

Some limitations exist in our systematic review. We only selected published studies written in English and involved studies from 2002 onwards; there may be studies in other languages or published before 2002 with vital findings. Also, all the studies only evaluated MDD at baseline and did not follow its evolution, which could have an impact on the connection between MDD and the risk of stroke. Additionally, most studies did not exclude the presence of any co-existing psychiatric illness that could have an influence on the MDD-stroke risk relationship. Moreover, most of the included studies were single-centered, which brings inherent selection bias into the data. Furthermore, because of diverse medical, behavioral, or societal responses to MDD or variable prevalence of other cardiovascular risk factors, the link between MDD and stroke may differ among countries and ethnicities. Consequently, it is imperative to undertake multicenter clinical trials in various populations to ensure generalisability and allow governments worldwide to put into place public health measures for MDD and stroke that are specific to the needs of their own country.

## Conclusions

To the best of our knowledge, this is the first systematic review to focus on the influence of major depressive disorder on stroke risk and mortality. The majority of the studies demonstrated a significant association between MDD and stroke risk and mortality. Moreover, multiple factors were identified that could potentially modify this relationship, but additional replication studies are required to draw definite conclusions about the possible effect modification. The apparent connection between MDD and stroke has medical and public health relevance, given the high incidence, prevalence, and financial burden of MDD and stroke in the general populace. Additionally, it is urgent to find new and unacknowledged factors of stroke risk as well as to treat existing stroke risk factors properly. There are numerous risk models and calculators for stroke. However, they only contain a small number of established risk factors. The predictive results of the stroke risk models may be improved by including additional factors, and managing these novel risk factors may further lower the risk of stroke. Therefore, it is imperative that further studies are conducted to confirm and validate this association between MDD and stroke while also elucidating the mechanism involved and investigating potential variables influencing this association. Moreover, additional studies must be done contrasting MDD with conventional stroke risk factors to determine its predictive usefulness compared to traditional risk factors. This will have a significant effect on clinical practice since the information provided by such research will help guide essential targets for stroke prevention.
